# Epigenetic regulation of transcription factor promoter regions by low-dose genistein through mitogen-activated protein kinase and mitogen-and-stress activated kinase 1 nongenomic signaling

**DOI:** 10.1186/s12964-016-0141-2

**Published:** 2016-08-31

**Authors:** Linda Yu, Kyle Ham, Xiaohua Gao, Lysandra Castro, Yitang Yan, Grace E. Kissling, Charles J. Tucker, Norris Flagler, Ray Dong, Trevor K. Archer, Darlene Dixon

**Affiliations:** 1 Molecular Pathogenesis Group, National Toxicology Program (NTP) Laboratory, Division of the NTP (DNTP), National Institute of Environmental Health Sciences (NIEHS), National Institutes of Health (NIH), U.S. Department of Health and Human Services (HHS), Research Triangle Park, North Carolina, 27709 USA; 2 Biostatistics and Computational Biology Branch, Division of Intramural Research (DIR), NIEHS, NIH, HHS, Research Triangle Park, North Carolina, 27709 USA; 3 Signal Transduction Laboratory, DIR, NIEHS, NIH, HHS, Research Triangle Park, North Carolina, 27709 USA; 4 Cellular and Molecular Pathology Branch, DNTP, NIEHS, NIH, HHS, Research Triangle Park, North Carolina, 27709 USA; 5 Chromatin and Gene Expression Group, Epigenetics and Stem Cell Biology Laboratory, DIR, NIEHS, NIH, HHS, Research Triangle Park, North Carolina, 27709 USA

**Keywords:** Epigenetic, Histone H3, Leiomyoma, MAPK_p44/42_, MSK1

## Abstract

**Background:**

The phytoestrogen, genistein at low doses nongenomically activates mitogen-activated protein kinase p44/42 (MAPK_p44/42_) via estrogen receptor alpha (ERα) leading to proliferation of human uterine leiomyoma cells. In this study, we evaluated if MAPK_p44/42_ could activate downstream effectors such as mitogen- and stress-activated protein kinase 1 (MSK1), which could then epigenetically modify histone H3 by phosphorylation following a low dose (1 μg/ml) of genistein.

**Results:**

Using hormone-responsive immortalized human uterine leiomyoma (ht-UtLM) cells, we found that genistein activated MAPK_p44/42_ and MSK1, and also increased phosphorylation of histone H3 at serine10 (H3S10ph) in ht-UtLM cells. Colocalization of phosphorylated MSK1 and H3S10ph was evident by confocal microscopy in ht-UtLM cells (*r* = 0.8533). Phosphorylation of both MSK1and H3S10ph was abrogated by PD98059 (PD), a MEK1 kinase inhibitor, thereby supporting genistein’s activation of MSK1 and Histone H3 was downstream of MAPK_p44/42_. In proliferative (estrogenic) phase human uterine fibroid tissues, phosphorylated MSK1 and H3S10ph showed increased immunoexpression compared to normal myometrial tissues, similar to results observed in in vitro studies following low-dose genistein administration. Real-time RT-PCR arrays showed induction of growth-related transcription factor genes, EGR1, Elk1, ID1, and MYB (cMyb) with confirmation by western blot, downstream of MAPK in response to low-dose genistein in ht-UtLM cells. Additionally, genistein induced associations of promoter regions of the above transcription factors with H3S10ph as evidenced by Chromatin Immunoprecipitation (ChIP) assays, which were inhibited by PD. Therefore, genistein epigenetically modified histone H3 by phosphorylation of serine 10, which was regulated by MSK1 and MAPK activation.

**Conclusion:**

Histone H3 phosphorylation possibly represents a mechanism whereby increased transcriptional activation occurs following low-dose genistein exposure.

**Electronic supplementary material:**

The online version of this article (doi:10.1186/s12964-016-0141-2) contains supplementary material, which is available to authorized users.

## Background

Uterine leiomyomas “fibroids” are the most common tumors found in the genital tract of both premenopausal and postmenopausal women [1]. Even though these tumors are benign, uterine leiomyomas have a significant impact on the reproductive health of women due to their high incidence and lack of proven treatment options other than surgery [2]. There is very little known about the etiology or pathogenesis of these tumors, although it is known that they are hormonally regulated and many growth factors upstream of MAPK appear to play a major role in their growth [3]. The role of certain environmental estrogens in the pathogenesis of fibroids remains to be elucidated [4].

Genistein is a soy-derived phytoestrogen that has been shown to be an anti-cancerous agent, and reported to have a stimulatory or inhibitory effect on cell proliferation depending on its concentration [5–7]. The plasma levels of genistein in humans ranges from 10 nM to 10 μM [8]. In previous in vitro experiments in our laboratory, we have found that a low concentration (1 μg/ml; 3.7 μM) of genistein, which is in the range of human exposures, stimulates growth of human uterine leiomyoma cells [7, 9]. Genistein is known for interacting with estrogen receptors alpha and beta (ERα and ERβ) [10]. Studies suggest that the effects observed with genistein and other estrogens, and classical ER binding is dependent on the ER type and content of the ER in target tissues or cells of interest [9, 11, 12]. It is thought that the effects observed in tissues whereby there is an abundance of ERα, as seen in the uterus and uterine cells, may be different from those observed in the prostate gland and ovary, in which ERβ is dominant [11, 12]. Therefore, the varying levels of ERα, or and ERβ within a given tissue or cell type are thought to dictate the responses of those tissues to estrogens or estrogen mimics [9, 10]. It is speculated that the tissue-specific effects observed in response to estrogens or estrogenic compounds may also be driven by the estrogen concentration, balance of ERα versus ERβ, and variation in transcription factors, coactivators and corepressors activated by ERα or ERβ [11, 12]. Estrogen also exerts biological effects through membrane-associated receptors, such as ERα36, ERα46 and G protein-coupled estrogen receptor 1, GPER1, to initiate nongenomic events leading to cell proliferation [13]. We have previously reported that our uterine leiomyoma cells express both ERα and ERβ receptors with higher expression levels of ERα [9, 14]. Also, we have reported that ERα is involved in transient nongenomic activation of ERK/mitogen activated protein kinase (MAPK) by genistein (1 μg/ml) via its early induction of ERα and IGF-IR associations, leading to uterine leiomyoma cell proliferation [9].

MAPKs are protein kinases (or enzymes) that convert stimuli into a wide range of cellular responses [15]. MAPK pathways regulate gene expression, mitosis, differentiation and proliferation [15, 16]. MSK1 (mitogen- and stress-activated protein kinase) is a kinase that is activated as a result of phosphorylation by MAPK_p44/42_ in cells [17]. Histone H3 is involved in the structural modification of chromatin in eukaryotic cells, and is also thought to play a role in the long-term regulation of genes in cells. MSK1 is downstream of MAPK [17], and activation of Histone H3 can occur as a result of MSK1 phosphorylation. Hyper-phosphorylation of histone H3 on serine 10 site may cause cell chromatin structural changes to open transcriptional factor promoter regions leading to enhanced gene transcription, the outcome of which is cell and stimulus dependent, and can range from cellular differentiation and cell proliferation to cell transformation and neoplasia [18–20].

In this study, an in vitro experiment was done to determine if genistein could affect Histone H3 phosphorylation via MSK1 by MAPK activation in immortalized human uterine leiomyoma (ht-UtLM) cells. We also used immunohistochemistry to determine the differential expression of MSK1 and Histone H3 in leiomyoma and patient matched myometrial tissues during the proliferative (estrogenic) menstrual cycle phase. An understanding of how genistein regulates epigenetic changes through MAPK/MSK1/H3 activation will help in revealing the biological effects of environmental estrogens and their nongenomic regulation of transcription factors and transcriptional activation in fibroids and other hormonally regulated disease conditions.

## Results

### Genistein stimulates activation of MSK1 and H3S10ph through MAPKp44/42 phosphorylation

**Fig. 1:**
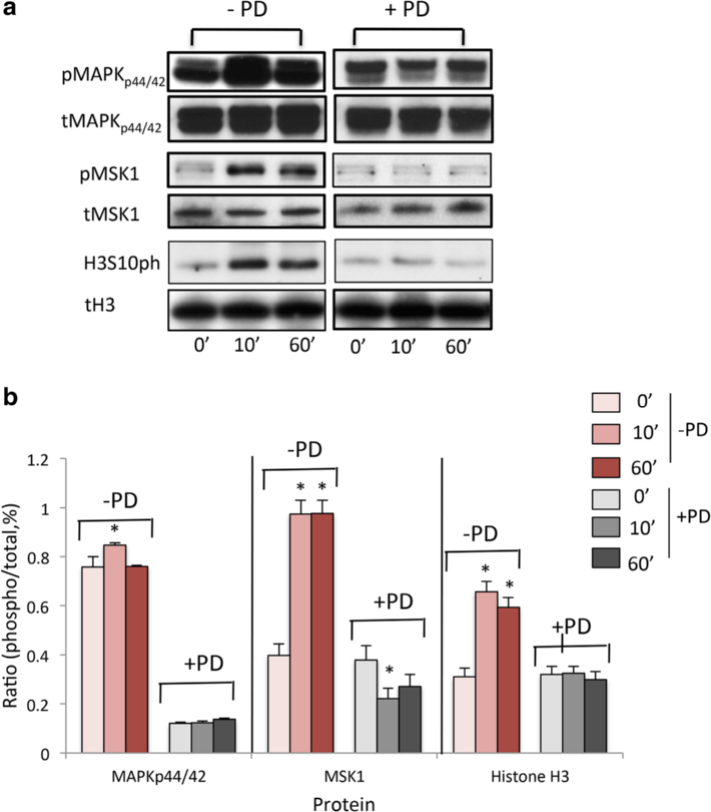
Differential expression of phosphorylated MAPK_p44/42_, MSK1, and H3 in ht-UtLM cells. **a** Western blots of total (t) and phosphorylated (p) MAPK_p44/42_, MSK1 and H3 (phosphorylated at serine 10; H3S10ph) proteins with PD (PD+) or without PD (PD-) followed by genistein (1 μg/ml) treatment for 0, 10 or 60 min. **b** Comparison of ratio of densitometric band intensities of phosphorylated/total (p/t, %) proteins in ht-UtLM cells. Data shows the mean+/-SEM of three independent experiments; *p < 0.05 versus 0 min

Previously, MAPK_p44/42_ was reported to be constitutively activated in human uterine leiomyoma cells [9], and MEK1 specifically phosphorylates MAPK_p44/42_ [21]. We examined whether the increased levels of phosphorylated MAPK_p44/42_ in ht-UtLM cells induced by genistein at 1 μg/ml could activate downstream MSK1 and lead to Histone H3 phosphorylation. We found there was increased expression levels of phosphorylated MAPK_p44/42_ in ht-UtLM cells, and there was also increased MSK1 and Histone H3 activation downstream of MAPK_p44/42_ following genistein exposure at 1 μg/ml at 10 min for phosphorylated MAPK _p44/42,_ and 10 and 60 min for phosphorylated MSK1 (pMSK1) and H3S10ph (by using a specific antibody targeting Histone H3 at phosphorylated serine 10 site only), respectively, in the cells (Fig. 1a and b). The increased phosphorylation of MAPK_p44/42_ induced by genistein in ht-UtLM cells was attenuated by a MEK1 inhibitor, PD, and the activation of MSK1 and H3S10ph downstream of MAPK_p44/42_ were also inhibited by PD (Fig. 1a and b), which would suggest that the phosphorylation of MSK1 and H3S10ph in ht-UtLM cells was mediated by pMAPK_p44/42_.

### Phospho-MSK1 and H3S10ph are highly expressed in tumors compared to myometrial tissues from women in the proliferative (estrogenic) phase of the menstrual cycle

**Fig. 2:**
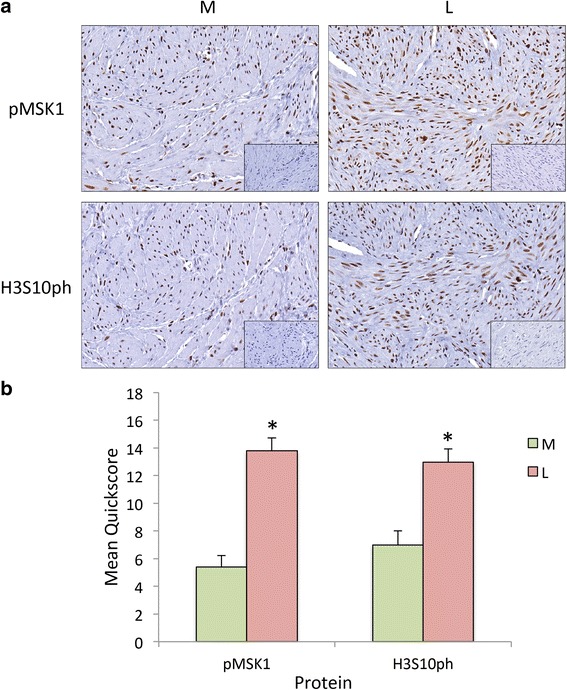
Increased immunolocalization of phosphorylated MSK1 and H3S10ph in leiomyoma (L) and myometrial (M) tissues. **a** The immunohistochemical staining was performed on L and matched M tissue samples from 16 patients to determine the phosphorylated (p) MSK1, and H3S10ph expression patterns. Inset: Negative control with normal rabbit IgG. **b** The bar graph shows the mean+/-SEM of the quickscores from 16 patient-matched samples in the proliferative phase of the menstrual cycle; *P < 0.001 L versus M tissue

The phospho-MSK1 was intensively expressed in all 16 patient leiomyoma and myometrial tissues (Fig. 2a), with much higher expression of phospho-MSK1 in the tumors compared to patient matched myometrial tissue samples (Fig. 2b). The H3S10ph was also highly expressed in leiomyoma and myometrial tissues (Fig. 2a), with an increased level in the tumor compared to patient matched myometrium (Fig. 2b). However, there were no differential expression of total MSK1 and Histone H3 between leiomyoma and myometrial tissues. Under the influence of estrogen in vivo, tissue samples showed an overall overexpression of activated MSK1 and H3S10ph similar to ht-UtLM cells exposed to a low-dose of genistein, an estrogen mimic.

### Increased colocalization of phosphorylated MSK1 and H3S10ph induced by genistein (1 μg/ml) in ht-UtLM Cells and the effects were abolished by PD

**Fig. 3:**
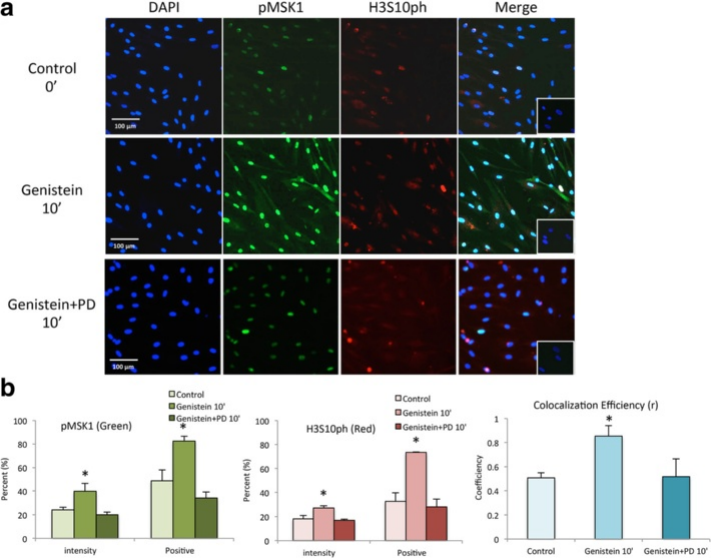
Colocalization of phosphorylated (p) MSK1 and H3S10ph in ht-UtLM cells treated with genistein (1 μg/ml). **a** The immunofluorescence staining was performed to detect Phospho-MSK1 (*green*) and H3S10ph (*red*) colocalization in ht-UtLM cells in the presence or absence of the PD inhibitor following genistein exposure at 0 min (control) and 10 min. Inset: Negative control with normal rabbit serum. **b** The intensity (%) and positive staining area (%) of phospho-MSK1 and H3S10ph induced by genistein, and the Pearson’s coefficiency of colocalization (r) of pMSK1 and H3S10ph colocalized signals. Data present the mean+/-SEM of percentage (%) of positive staining area and intensity from scanned images; *p < 0.05 treated 10 min versus non-treated 0 min. Blue = DAPI nuclear stain. Bar = Magnification in μm

Due to increased expression of pMAPK_p44/42,_ pMSK1and H3S10ph by western blotting, we were interested in determining whether phospho-MSK1 and H3S10ph were colocalized following genistein administration using immunofluorescence staining and confocal microscopy. As shown in Fig. 3a, confocal microscopy revealed that genistein rapidly increased phosphorylation levels of MSK1 and H3S10ph in the nuclei of ht-UtLM cells at 10 min, and these effects were abrogated in the presence of PD pre-treatment. Similar effects for MSK1 and H3S10 activation were seen up to 60 min and were also inhibited by PD in ht-UtLM cells (see Additional file 1). Confocal microscopy further indicated that there was an overlapping of green (pMSK1) and red (H3S10ph), and there was enhanced phospho-MSK1 and H3S10ph colocalization (*r* = 0.8533 versus control, *r* = 0.5067) in the nuclei of ht-UtLM cells following 10 min of genistein administration, and decreased colocalization (*r* = 0.516) after PD pre-treatment (Fig. 3b).

### Genistein enhances cell proliferation-related transcription factor gene expression

**Fig. 4:**
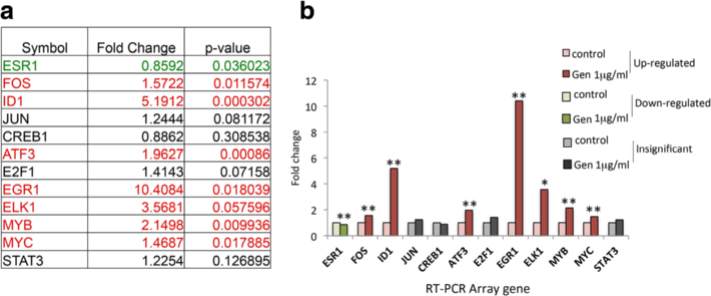
Differential expression of transcription factor genes induced by genistein (1 μg/ml) in ht-UtLM cells at 24 h. **a** The fold changes by Real-Time PCR array for ht-UtLM cells. **b** Plot of fold changes of transcription factor genes in response to genistein treatment in ht-UtLM cells. Data represent the mean+/-SEM of three independent experiments. **p ≤ 0.0003 – 0.0360 versus control (no treatment); *p = 0.0575

To identify the transcription factors possibly involved in genistein modulation of cell proliferation previously observed in our earlier studies in UtLM cells treated with genistein (1 μg/ml) but not in UtSMC [9], we used a custom designed 384-well real-time RT PCR array plate coated with 12 predispensed transcriptional factor gene-specific primer sets (SABiosciences) and the GAPDH housekeeping gene as a control. The 12 genes were selected based on their up- or down regulation following genistein treatment using a Human Transcription Factors RT^2^ Profiler PCR Array (Qiagen) containing 84 genes (see Additional file 2). Total RNA isolated from ht-UtLM cells stimulated with genistein (1 μg/mL) for 24 h was used for the array. We identified increases (P ≤ 0.0003-0.0575) in expression of seven transcription factor genes, and decreased expression of one transcription factor gene (ESR1) in genistein stimulated ht-UtLM cells compared to non-stimulated cells (Fig. 4a and b). Four transcription factor genes had a > 2-fold change: EGR1 (10.41), ELK1 (3.57), ID1 (5.19), and cMyb (2.15) (Fig. 4a), which would be further confirmed by western blot. These results suggest that genistein may have modulated cell growth through enhanced specific transcriptional regulatory mechanisms in ht-UtLM cells. As shown in Fig. 4b, EGR1 expression in genistein-stimulated ht-UtLM showed the highest significant increase in mRNA levels, with modest, but significant increases in mRNA levels of ID1 and cMyb, and a marginally significant increase in ELK1 compared with unstimulated cells.

### Increased protein expression of cell proliferation related transcription Factor was confirmed

**Fig. 5:**
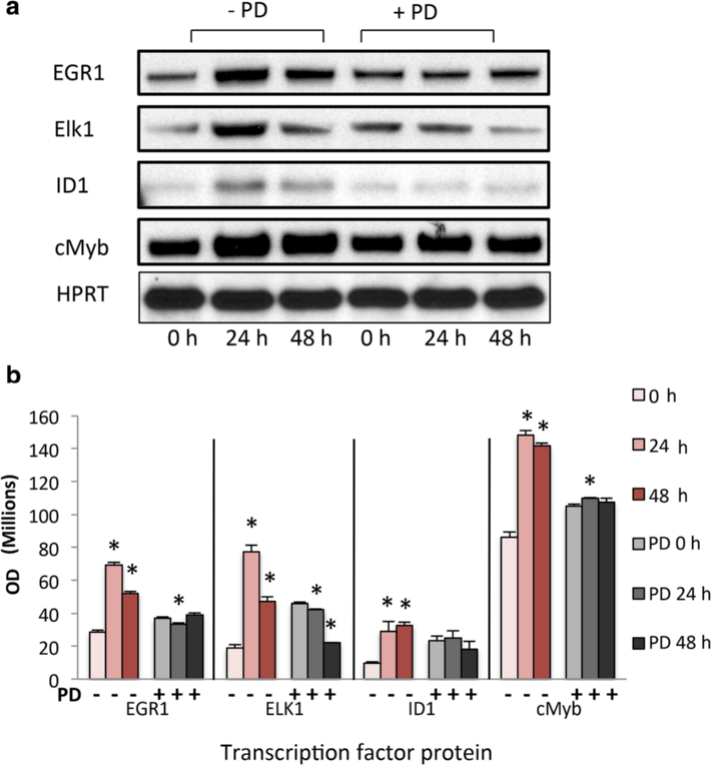
Differential expression of transcription factor proteins induced by genistein (1 μg/ml) in ht-UtLM cells for 0, 24 and 48 h*.* **a** Western blot of EGR1, Elk1, ID1 and MYB proteins in ht-UtLM cells stimulated with genistein with or without PD. Hypoxanthine-guanine phosphoribosyltransferase (HPRT) served as loading control. **b** Comparison of band intensities of transcription factor proteins in ht-UtLM cells. Data represent the mean+/-SEM of three independent experiments. *p < 0.05 versus 0 h

We next confirmed EGR1, Elk1, ID1, and cMyb protein expression in ht-UtLM cells at different time points. Total cell lysates obtained from cells stimulated with genistein (1 μg/mL) for 0, 24, and 48 h with or without PD were analyzed by western blotting for EGR1, Elk1, ID1, and cMyb expression. The cells stimulated with genistein had a significant increase in protein levels of EGR1, Elk1, ID1 and cMyb expression compared with unstimulated cells (Fig. 5). These results suggested that genistein could modulate cell proliferation-related transcription factor genes through specific transcriptional regulatory mechanisms in ht-UtLM cells.

### Genistein enriches the promoter region of transcriptional factors mediated by histone H3 phosphorylation

**Fig. 6:**
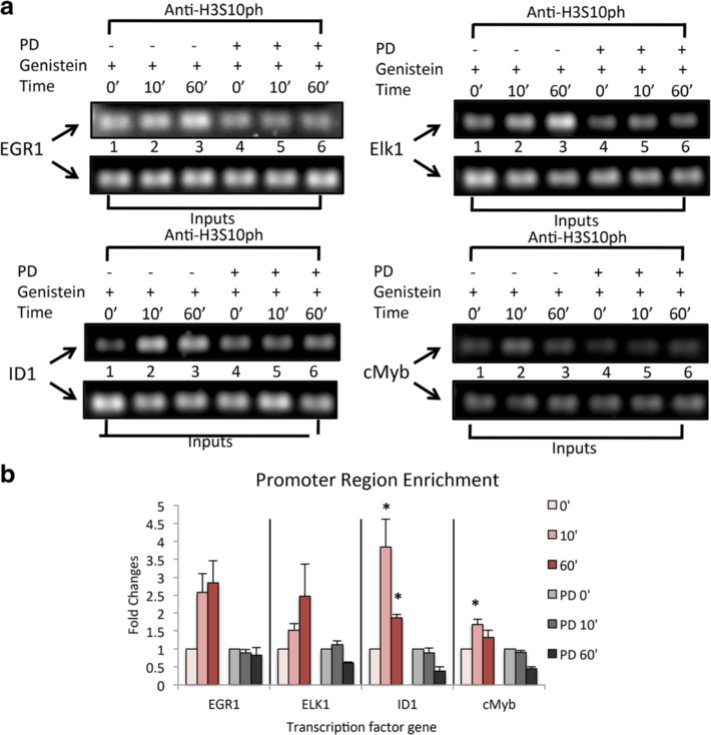
Differential expression of promoter regions of transcription factor genes in ht-UtLM cells with PD or without PD followed by genistein (1 μg/ml). **a** DNA gel image of promoter regions of transcription factors in ht-UtLM cells (DNA captured by H3S10ph antibody) in ChIP assay. **b** Plot of fold changes of enrichment of promoter regions of transcription factor genes in response to genistein treatment in ht-UtLM cells with or without PD. Data represent the mean+/-SEM of three independent experiments. *p < 0.05 versus 0 min

To further determine whether the histone H3ser10 phosphorylation site was associated with the promoter regions of genistein-induced transcription factors, EGR1, Elk1, ID1, and cMyb following genistein exposure in ht-UtLM cells, the promoter regions of the above transcription factors were examined using ChIP assays. The primers as shown in Table 1 were picked through searches of the four transcription factor gene promoter regions in databases. The gene promoter regions that co-precipitated with anti-H3S10ph antibody were determined by qRT-PCR. Increased levels of EGR1, Elk1, ID1 and cMyb promoter regions were observed; however, only the ID1 and cMyb promoter region enrichments were statistically significant. EGR1 and Elk1 promoter region enrichments were not statistically significant and this may have been due to variations in fold changes among the triplicates of ht-UtLM cells treated with genistein (Fig. 6). PD inhibited the enrichment of all four of these transcription factor promoter regions. These results suggest that the enrichment of the promoter regions following genistein administration was induced by phosphorylation of histone H3 at serine 10 and mediated by MAPK_p44/42,_ with subsequent MSK1 activation leading to gene transcription.

**Table 1:** Primers for the ChIP Assays

Primer Name	Sequence
EGR1-F	CCCTCACCACAAGGACCATT
EGR1-R	AAGCTGATTGCCCCAAGAGA
ELK1-F	CCCCCCATCCTCAGACATTA
ELK1-R	AAGAAGGGCCATGTGACTCAA
ID1-F	GCAGAAGGCTCCTTTACTTTTCC
ID1-R	GTTATCAGCAGGTTCCGTTTTCC
cMyB-F	CACTTCACAAAGGGTTCAGATACTCA
cMyB-R	CTGGCAAGATTCCCAATTGC

## Discussion

In our previous studies, we have found that genistein at a concentration of 1 μg/ml stimulates proliferation of ht-UtLM cells [7]. We also reported that genistein at 1 μg/ml could directly activate classic ER gene transcription, upregulate estrogen-responsive genes, and is involved in transient nongenomic activation of ERK/mitogen-activated protein kinase (MAPK_p44/42_) via its early induction of ERα and IGF-IR associations, leading to cell proliferation [9]. In this study, we further evaluated if MAPK_p44/42_ could activate a downstream effector, such as MSK1, which may perhaps epigenetically modify histone H3 by phosphorylation following a low dose (1 μg/ml) of genistein exposure in ht-UtLM cells.

A wide range of genes has been shown to be upregulated or downregulated in uterine tissue of rats treated with genistein [22]. A number of studies have shown that tumorigenesis genes are transcriptionally silenced through modifications of promoter-associated histones as functions of expression of MAPK-mediated tumor progression [23–25]. Upregulated transcription by an epigenetic mechanism can involve phosphorylation of histone H3 via the activation of tumorigenic MAPK pathways [26]. Although the mechanisms by which these pathways regulate histone phosphorylation remains unknown, understanding the molecular link(s) between these pathways and the regulation of histone modification would provide new insights into the molecular basis of human uterine leiomyoma related alterations of gene expression through chromatin remodeling induced by estrogen or environmental estrogen mimics, like genistein.

In this study, we demonstrated that the MAPK_p44/42_/ERK1/2 pathway can be activated by genistein in human uterine leiomyoma cells and lead not only to elevated levels of phospho-MAPK_p44/42_ but also to increased levels of downstream nucleosome effectors phosphorylated MSK1 and H3S10ph, and their colocalization. It has been proposed that histone H3 phosphorylation might result in a greater exposure of histone H3 to MSK1 [24]. The intensity and duration of signaling of MSK1 and histone H3 upon genistein stimulation correlates well with the activated MAPK_p44/42_ in the cells, which might indicate a higher level of chromosome dynamics [26] mediated by MAPK, and a more open chromatin structure compared to the untreated cells. The specificity of MAPK regulation of MSK/histone H3 expression was further confirmed through PD98059 suppression of MAPK_p44/42_ expression in these cells. Western blot analysis of total cell lysates obtained from cells treated with PD revealed inhibition of MAPK_p44/42_expression in ht-UtLM cells leading to abrogation of phosphorylation of MSK1 and H3S10ph induced by genistein.

Phosphorylation of MSK1 and histone H3 is an epigenetic mechanism altering gene expression [18]. Epigenetic alterations, including DNA methylation and histone acetylation and phosphorylation, might independently regulate gene expression of DNA sequences [27]. Alterations in epigenetic programming in the ht-UtLM cells may alter the timing of induction of genes by changing the chromatin landscape of MSK1 and histone H3 target genes upon genistein exposure. In the case of MAPK signaling in ht-UtLM cells, genistein increased phospho-MAPK_p44/42_ levels resulting in a high predilection for MSK1 and histone H3 phosphorylation at serine10 site that induced epigenetic changes that led to enrichment of promoter regions of immediate early response genes known to correspond to cell proliferation and tumorigenesis [25]. It was reported that MSK1 is recruited to promoters of target genes by transcription factors such as Elk1 and the progesterone receptor [19, 20, 28]. In the cells there is a change in transcription factors composition and activity, which may alter the dynamics of MSK1 recruitment to target promoters to activate histone H3 leading to chromatin structure alterations [18] and in turn increase target promoter expression levels during tumorigenesis.

Human gene expression is largely controlled at the level of transcription and post transcription. Involvement of transcriptional factors in different signal transduction pathways determines the level of expression of a specific gene. Abnormalities in transcription factors are associated with a number of human diseases [29]. The real-time PCR array, with twelve potential transcription factors from cell proliferation-related transcription factor gene families in this study, suggested that the enhanced activity of MSK1 and elevated levels of phosphorylated histone H3 contributed to significantly higher levels of four transcription factor genes (EGR1: 10.41-fold; Elk1: 3.57-fold; ID1: 5.19-fold; and cMyb: 2.15-fold) in genistein treated ht-UtLM cells relative to those cells without treatment. The differences in transcription factor expression before and after genistein induction may be a consequence of changes in the activated MSK1 and H3S10ph recruiting more MSK1 to the promoter regions, and further phosphorylation of histone H3 resulting in epigenetic changes in gene expression programming and genomic instability [28] in ht-UtLM cells.

To determine if the promoter regions of transcription factors are opened more due to chromatin structural alterations caused by histone H3 phosphorylation, we used anti-H3S10ph antibody, with EGR1, Elk1, ID1, and cMyb (>2-fold) promoter sequences as targets, in ChIP assays and found enrichment of the above transcription factor promoter regions after genistein treatment, restricting the target input to the promoter regions present in the ht-UtLM cells compared to untreated cells. The inhibition by PD, a MAPK specific inhibitor, attenuated the genistein induced MSK1 and histone H3 activation, and paralleled the abrogation of the enrichment of the promoter regions. Confirmation studies by western blotting revealed that PD also reduced transcript levels of EGR1, Elk1, ID1, and cMyb. These four transcription factor proteins were highly expressed in the cells treated with genistein compared to non-treated cells, and this highly expressed level was attenuated by PD, which indicated that the MAPK pathway played a critical role in the chromatin remodeling process that resulted in higher levels of transcription factor expression possibly leading to increased cell proliferation as observed in our previous studies [7, 9].

EGR1, early growth response-1, is a Cys2-His2-type zinc-finger transcription factor. It is involved in a number of biological processes including cellular growth, proliferation, differentiation and matrix re-modeling. In response to smooth muscle injury, EGR1 may activate matrix remodeling genes and profibrotic genes such as TGF-β, PDGF, and Collagen 1 genes [29, 30], which have all been suggested as etiological factors involved in the pathogenesis of human uterine leiomyomas. The transcriptional factor of Elk1 belongs to the ternary complex factor (TCF) subgroup of the Ets family, which is a target of the MAPK cascade [31]. It was found in our previous gene array studies that the genes involved in IGF-IR/MAPK signaling were upregulated in UtLM cells by estrogen, including MAPKs, MAPK kinases, transcriptional factor Elk1 and others all involved in cell cycle progression, proliferation, and cell survival [30]. The upregulation of Elk1 induced by genistein, in this study further confirms the involvement of the MAPK/MSK/histone H3 pathway and cell proliferation transcription factors in hormonally driven leiomyoma development. There is evidence that Elk1 bridges the gap between SRF-mediated gene transcription, EGR1 and the Raf/MEK/ERK pathway to promote cell proliferation [29]. ID1, the helix-loop-helix transcription factor, also enhances cell proliferation. Its expression has been associated with the induction of tumor cell growth and promotion of cell survival. ID1 protein is frequently overexpressed in over 20 types of cancer, supporting its role in the tumorigenesis of a wide range of tissues [32]. ID1 mRNA levels are significantly increased in uterine cervical cancers [33]. Also, there is a report showing that the expression of ID1 is tightly regulated by estrogen in the mouse uterus [34]. Our study is the first study to reveal ID1 expression in human uterine leiomyoma cells downstream of histone H3 phosphorylation induced by genistein. Therefore, ID1 might work in uterine leiomyoma tumorigenesis through histone H3 activity. As a key regulator of proliferation, differentiation and cell fate, the cMyb transcription factor regulates the expression of hundreds of genes and in turn is regulated by numerous pathways and protein interactions. One of the most unique features of cMyb is the extremely complex nature of its regulation. The expression of the cMyb gene is regulated at several levels, including a sophisticated control of transcriptional elongation and that may be regulated by transcription factors or by the estrogen receptor alpha [35]. The increased expression of cMyb in ht-UtLM cells after genistein treatment indicates that its upregulation may be mediated through an estrogen receptor-MAPK/MSK1/histone H3 pathway.

## Conclusions

**Fig. 7:**
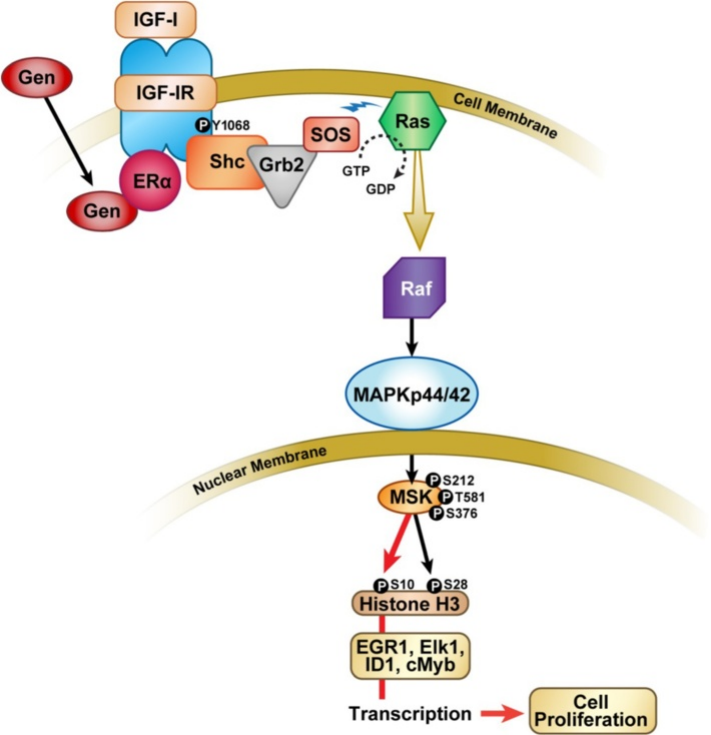
Schematic illustration of MAPK/MSK1/Histone H3 pathway on target gene transcription. The MAPK/MSK1/Histone3 pathway induced by genistein via nongenomic transient interactions of ERα with receptor tyrosine kinases (IGF-IR) and activation of MAPK_p44/42_/ERK1/2 [9], with recruitment and phosphorylation of MSK1 and activation of Histone H3 leading to enrichment of promoter regions of transcription factors EGR1, Elk1, ID-1 and cMyb and the genes transcription in human uterine leiomyoma (ht-UtLM) cells

In conclusion, we demonstrate the epigenetic regulation of transcription factor promoter regions by MAPK_p44/42_ through MSK1 and histone-H3 activation in human uterine leiomyoma cells exposed to genistein (1 μg/ml) (Fig. 7). These genistein-induced transcriptional activities may contribute to upregulation of genes involved in the increased proliferative properties of these cells observed in our earlier studies. The genistein exposed cells had changes in the histone H3 phosphorylation epigenetic program, which may have contributed to alterations in induced transcription factor gene expression responses observed. Moreover, the increased activity of MSK1 through MAPK phosphorylation in the cells contributes to the enhanced steady state levels of histone H3 phosphorylation. These changes in histone modification likely contribute to a less condensed chromatin structure in ht-UtLM cells. It has been reported that MSK1 and histone H3 serine 10 phosphorylation are required for tumor promoter induced cell proliferation and transformation [25, 36]. The contribution of MSK1 activity and histone H3 phosphorylation to chromosomal dynamics in human uterine leiomyoma cells might be a therapeutic target or intervention site for clinical fibroids stimulated to grow by endogenous and/or environmental estrogens.

## Methods

### Cells

Ht-UtLM cells were previously established in our lab [14] and were grown to 80 % confluency in Minimum Essential Medium (MEM) (Gibco) Containing supplements. The medium was later switched to charcoal/dextran stripped Fetal Bovine Serum (FBS) (Sigma) in Dulbecco’s modified Eagle Medium/Nutrient Mixture F-12 Ham (DMEM-F12) (Gibco) without phenol red for 24 h prior to treatment with 1 μg/ml of genistein (Sigma) and/or 50 μg/ml of MEK1 inhibitor PD98059 (PD) (Cell Signaling), with pretreatment of PD for 2 h.

### Western Blot

Cell lysates containing total protein of cells were harvested in RIPA buffer [9] at 0, 10, and 60 min after treatment with genistein. The lysate (30 μg/well) samples were loaded onto a 4-12 % Bis Tris Gel and then transferred to a PVDF membrane. Western blotting was performed as previously described [37]. The primary antibodies (incubated overnight at 4 °C) used were: rabbit polyclonal anti-MAPK_p44/42_ and anti-phospho-MAPK_p44/42_ (Cell Signaling), rabbit polyclonal anti-MSK1 (Santa Cruz Biotechnology), rabbit polyclonal anti-Phospho-MSK1 (Cell Signaling), rabbit polyclonal anti-Histone-H3 (Cell Signaling), and rabbit monoclonal anti-Phospho-Histone-H3Ser10 (Cell Signaling). The secondary antibodies used were: donkey anti-rabbit horseradish peroxidase conjugated antibody (GE Health Care) 1 h at room temperature (r.t.), rabbit polyclonal anti-EGR1, Elk1, and cMyb (Cell Signaling) and rabbit polyclonal anti-ID1 (Santa Cruz) which all incubated overnight at 4 °C to confirm real-time PCR array results as to whether the transcriptional factor gene expression levels induced by genistein also lead to the protein expression level changes. Detection was done for 1 min using ECL Western blotting reagents (GE Health Care). Comparison of ratio (%) of densitometric band intensities (FluorChem^TM^, Alpha Innotech) of phosphorylated to total proteins in control and ht-UtLM cells following genistein treatments were done.

### Immunhistochemistry

Uterine leiomyoma and patient matched myometrial tissues were obtained from 16 patients ranging from 41 to 49 years of age in the proliferative (estrogenic) phase of the menstrual cycle. The tissue slides were deparaffinized, rehydrated, peroxidase blocked, and antigen retrieved [38]; then the tissues were incubated with specific primary antibodies (overnight at 4 °C): anti-phospho-MSK1 and anti-H3S10ph (mentioned earlier in the Western blotting section), or with non-immune rabbit serum (negative control) (Jackson Immuno Research) at the same concentration as the primary antibodies. The secondary antibody (1 h at r.t., Vector Laboratories), label (Vector) and chromogen, 3,3’ diaminobenzidine tetrahydrochloride (DAB) (Dako) were applied. Lastly, the tissues were rehydrated and coverslipped. Slides were then scanned using the Aperio® Scanscope XT Digital Slide Scanner (Aperio Technologies). A semiquantitative numeric score incorporating overall percent of positive immunohistohemical staining and intensity of imunostaining was determined for leiomyoma and myometrial samples using the multiplicative quickscore method described by Detre et al. [39] and used by our lab previously [40, 41]. The intensity of staining for each tissue was categorized as negative = 0, weak = 1, moderate = 2, or intense = 3. This number was multiplied by a number generated to indicate the percent of tissue showing positive immunostaining (0-4 % = 1; 5-19 % = 2; 20-39 % = 3; 40-59 % = 4; 60-79 % = 5; 80-100 % = 6) to generate a quickscore number.

### Immunofluorescence (Confocal Microscopy)

Immunofluorescence staining was performed to detect phospho-MSK1 and H3S10ph colocalization at 0, 10, and 60 min in ht-UtLM cells following genistein treatment (1 μg/ml) with or without PD inhibition. The cells were grown on glass-bottom dishes and fixed with ice cold 100 % methanol (Avantor Performance), permeabilized with 0.2 % Triton X-100 (Sigma), and blocked with 5 % BSA (Sigma) and 0.1 % gelatin (Sigma) in PBS. The cells were incubated overnight with primary phospho-MSK1 rabbit polyclonal (Cell Signaling) and phospho-Histone H3ser10 rabbit monoclonal antibodies (Alexa Flour 594 conjugated, Cell Signaling), followed by the secondary Alexa Fluor 488 goat anti-rabbit phospho-MSK1 secondary antibody (Invitrogen) and DAPI (Invitrogen). Non-immune rabbit serum (Jackson Immunoresearch) at the same concentration as the primary antibodies, served as negative controls. Confocal images were taken on a Zeiss LSM780 (Carl Zeiss). The 561 nm laser line from a DPSS laser at 5 % power was used for excitation of H3S10ph labeled with Alexa594. Subsequently, the 488 nm line of an Argon laser was used at 2.0 % power to excite the pMSK1 labeled Alexa488 with a 491 - 553 nm band pass filter that collected the emission signal. Image analysis was done with Metamorph (Molecular Devices) using the Multi Wavelength Cell Scoring Application. With this application Metamorph uses the DAPI signal to define the nucleus and then it is able to extract the average intensity inside the nucleus for the green and red channels. Additionally, thresholds were set (15 gray values for green channel, 10 gray values for red channel) that were used to define a cell as positive or negative for that specific channel (percent of positive cells over total cells). The average Pearson coefficient of colocalization was obtained to determine the strength and direction of the linear relationship between phospho-MSK1 and H3S10ph signals that is defined as the signal values divided by their standard deviations.

### Transcriptional Factor real-time PCR array

Total cellular RNA was extracted from the cells using Qiagen RNeasy Mini Kit (SABiosciences) followed by the RT^2^ First Strand C-03 Kit (SABiosciences) to remove any residual contamination of the RNA samples with 2 μg purified RNA per treatment condition. The template combined with the RT^2^ SYBR green/ROX qPCR mix (SABiosciences) was loaded into a custom designed 384-well array plate coated with 12 pre-dispensed transcriptional factor gene-specific primer sets (SABiosciences) with four repeats of each treatment condition, and three independent studies on the plate, and processed on a TaqMan ABI Prism 7900 Sequence Detector System (Applied Biosystems) according to the RT^2^ Profiler PCR Arrays (SABiosciences) manufacturer’s protocol. The data analysis was based on the ΔΔCt method with normalization to GAPDH (http://www.sabiosciences.com/pcrarraydataanalysis.php). If the fold changes of treatment group ΔΔCt over non-treatment group ΔΔCt were larger or equal to 2-fold, the transfection factor would be chosen for further confirmation studies.

### Chromatin Immunoprecipitation (ChIP) Assay

A ChIP assay was performed using SimpleChIP Plus Enzymatic Chromatin IP Kit Cell Signaling) following the protocol outlined by the manufacturer with minor modifications. Briefly, protein and associated chromatin in cells treated with genistein were linked by 1 % formaldehyde for 10 min. After washing with cold PBS, cells were collected by centrifugation and resuspended in SDS lysis buffer (50 μM Tris-HCl, 10 μM EDTA, 1 % SDS, 1 μM PMSF, 10 μg/ml aprotinin and leupeptin) and incubated for 10 min on ice. The DNA-protein complexes (chromatin-protein) were enzyme digested and sonicated into 200–1000 bp fragments. The fragments were immunoprecipitated with 2 μg of antibody against H3S10ph (Cell Signaling) and the DNA fragments associated with H3S10ph were selectively immunoprecipitated, and the associated DNA fragments were purified and subjected to RT-PCR for enrichment of the transcriptional factor promoter regions of EGR1, ELK1, ID1, and MYB (cMyb) using SYBR Premix. The primers (Table 1) were picked by website searches: (http://www.ncbi.nlm.nih.gov/nuccore/) and (http://www.ncbi.nlm.nih.gov/pubmed/). The primers were located in the promoter region upstream of the TATA or TATA-like box.

### Statistical analysis

To demonstrate whether statistically significant differences existed among different groups of treated or not treated with or without PD, the two-sided t-tests were used to compare 10’ to 0’ and 60’ to 0’, or 24 to 0 h and 48 to 0 h for western blot band intensities. The Mann-Whitney tests [42] were used to compare 10’ to 0’ and 60’to 0’ for western blot band intensities, and were also used to compare treated 10’ and 60’ to not treated (0’) for staining intensity, positive area and colocalization efficiency for confocal Immunofluorescence images. In the immunohistochemistry studies, each patient contributed a tumor sample and a normal tissue sample, therefore, Wilcoxon signed rank tests [43] were used to compare mean staining % between tumor and normal tissue. For all target transcription factor genes in the real-time RT-PCR studies, the statistical analysis was performed on the normalized values with housekeeping genes using the ΔΔCt method. Because all 0’ fold changes were 1.000, one-sample t-test [44] was used to test that the 10’ and 60’ fold changes were equal to 1.000.

## Additional files

Additional file 1: Colocalization of phosphorylated (Phospho-) MSK1 and H3S10ph in ht-UtLM cells treated with genistein (1 μg/ml). The immunofluorescence staining was performed to detect Phospho-MSK1 (green) and H3S10ph (red) colocalization in ht-UtLM cells in the presence or absence of the PD inhibitor following genistein exposure at 0 min (control; shown in Fig. 3a) 10 min. (see Fig. 3a), and 60 min. (60 min. time point was originally part of data set shown in Fig. 3a). (TIF 1048 kb) Additional file 2: Differential expression of cell proliferation related transcription factor genes induced by genistein (1 μg/ml) in ht-UtLM cells at 24 h using a Human Transcription Factors RT^2^ Profiler PCR Array (Qiagen) containing 84 genes. (TIF 1131 kb)

## Abbreviations

ChIP: Chromatin immunoprecipitation
EGR1: Epidermal growth factor receptor 1 gene
Elk1: Member Of ETS oncogene family
ERα: Estrogen receptor alpha
ERβ: Estrogen receptor beta
H3S10ph: Histone H3 phosphorylated at serine10
ht-UtLM: Human uterine leiomyoma
ID1: Inhibitor of DNA Binding 1
IGF 1: Insulin-like growth factor 1
IGF-IR: Insulin-like growth factor 1 receptor
MAPK_p44/42_: Mitogen-activated protein kinase (p44/42)
MSK1: Mitogen- and stress-activated protein kinase 1
MYB (cMYB): V-Myb Avian Myeloblastosis
PD: PD98059
PDGF: Platelet-derived growth factor
qRT-PCR: Quantitative real time polymerase chain reaction
RT-PCR: Real time polymerase chain reaction
