# Supplementary figures and images for: Epigenetic regulation of transcription factor promoter regions by low-dose genistein through mitogen-activated protein kinase and mitogen-and-stress activated kinase 1 nongenomic signaling
Source: Cell Commun Signal. 2016 Aug 31;14(1):18. doi: 10.1186/s12964-016-0141-2 (PMC5007815; doi:10.1186/s12964-016-0141-2)

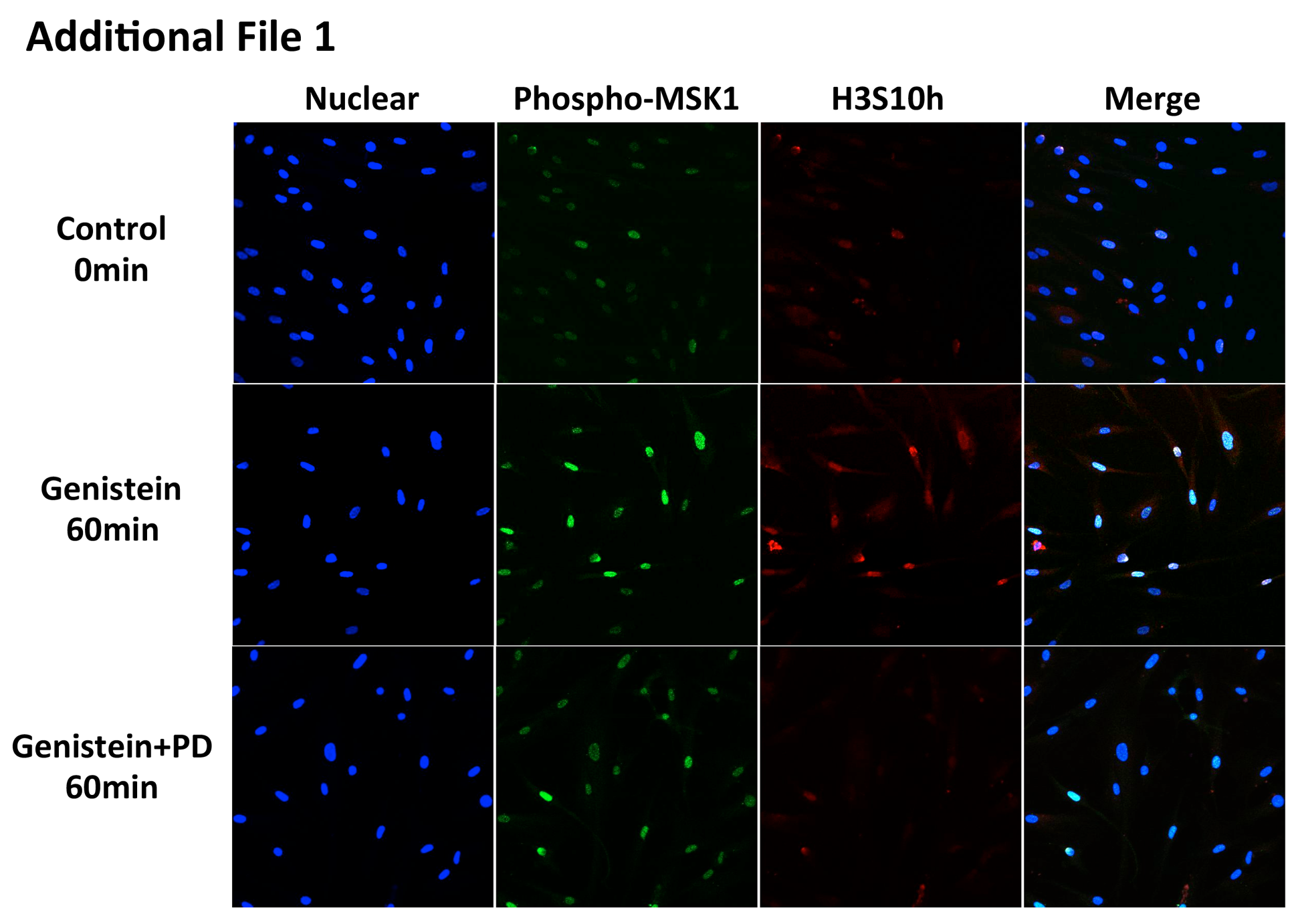

Supplement: Additional file 1: — Colocalization of phosphorylated (Phospho-) MSK1 and H3S10ph in ht-UtLM cells treated with genistein (1 μg/ml). The immunofluorescence staining was performed to detect Phospho-MSK1 (green) and H3S10ph (red) colocalization in ht-UtLM cells in the presence or absence of the PD inhibitor following genistein exposure at 0 min (control; shown in Fig. 3a) 10 min. (see Fig. 3a), and 60 min. (60 min. time point was originally part of data set shown in Fig. 3a). (TIF 1048 kb) [file 12964_2016_141_MOESM1_ESM.tif]

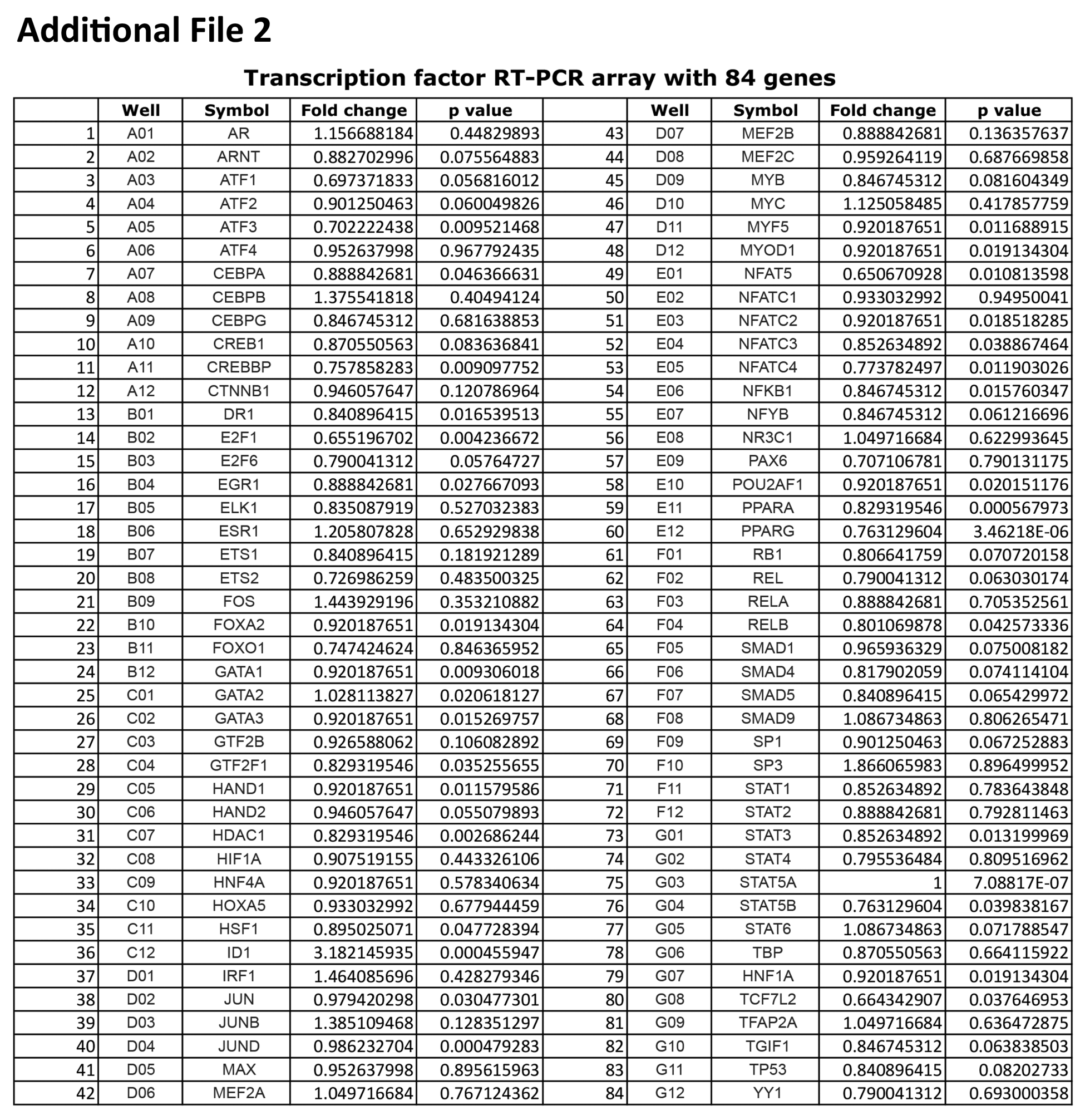

Supplement: Additional file 2: — Differential expression of cell proliferation related transcription factor genes induced by genistein (1 μg/ml) in ht-UtLM cells at 24 h using a Human Transcription Factors RT2 Profiler PCR Array (Qiagen) containing 84 genes. (TIF 1131 kb) [file 12964_2016_141_MOESM2_ESM.tif]
